# Exploring the Association Between Behavioral Determinants and Intention to Use a Chatbot-Led Parenting Intervention by Caregivers of Adolescent Girls in South Africa: Cross-Sectional Study

**DOI:** 10.2196/76992

**Published:** 2025-09-22

**Authors:** Maria Da Graca Ambrosio, Seema Vyas, Juliet Stromin, Shallen Lusinga, Paula Zinzer, Kanyisile Brukwe, Zamakhanya Makhanya, Hlengiwe Gwebu, Anne Schley, Laurie Markle, David Stern, Chiara Facciolà, G J Melendez-Torres, Frances Gardner, Jamie M Lachman

**Affiliations:** 1 Department of Social Policy and Intervention, University of Oxford, 32 Wellington Square, Oxford, OX1 2ER, United Kingdom, +44 01865270325; 2 Centre for Social Science Research, University of Cape Town, Cape Town, South Africa; 3 Clowns Without Borders, Cape Town, South Africa; 4 Department of Nursing and Public Health, University of Fort Hare, East London, South Africa; 5 mothers2mothers, Mbombela, South Africa; 6 Global Parenting Initiative, Oxford, United Kingdom; 7 IDEMS International Community Interest Company, Reading, United Kingdom; 8 Faculty of Health and Life Sciences, University of Exeter, Exeter, United Kingdom

**Keywords:** digital health, engagement, adolescent health, behavioral determinants, chatbot-led parenting intervention.

## Abstract

**Background:**

While digital innovation, including chatbots, offers a potentially cost-effective means to scale public health programs in low-income settings, user engagement rates remain low. Barriers to participant engagement (eg, perceived difficulty of use, busyness, low levels of digital literacy) may exacerbate inequality when adopting digital-only interventions as alternatives to in-person programs.

**Objective:**

This cross-sectional study nested within a 2×2 clustered factorial trial that followed the Multiphase Optimization Strategy principles investigated the relationship between behavioral determinants (ie, human and socioeconomic characteristics that facilitate the use of digital health interventions [DHIs]) and caregiver intention to use a digital public health intervention, ParentText, an open-source, rule-based parenting chatbot designed to promote positive parenting, improve adolescent health, and reduce risky behaviors.

**Methods:**

Caregivers of adolescent girls (10‐17 years; N=1034 caregivers) were recruited by implementation partners from a community-wide project aimed at HIV prevention in two districts of Mpumalanga, South Africa. A Digital Health Engagement Model was adapted from the technology acceptance model, the PEN-3 model theoretical frameworks, and the Theory of Planned Behavior to investigate the relationship between behavioral determinants and the intentions of caregivers to engage in ParentText. Community facilitators administered baseline surveys to caregivers during intervention onboarding. Regression models tested associations between behavioral determinants (ie, perceived ease of use, perceived usefulness, attitude, hedonic motivation, habit, price value, and social influence) and intentions of caregivers to use the parenting chatbot. Interaction effects were explored to examine whether individual-level sociodemographic and psychosocial characteristics moderate associations between overall behavioral determinants and intentions to use the chatbot.

**Results:**

Caregivers reported a mean of 2.85 (SD 0.79) and 2.90 (SD 0.72) out of a maximum score of 4 regarding their intention to use their mobile data and to continue using ParentText in the future, respectively. Overall behavioral determinants predicted by 76% (odds ratio 1.76, 95% CI 1.72‐1.81) the intentions of caregivers to spend mobile data and by 85% (odds ratio 1.85, 95% CI 1.81‐1.90) their intentions to use ParentText in the future. Moderator analysis suggested the interaction effects of age, paternal absence, financial efficacy, and stress on the relationship between overall behavioral determinants and intention outcomes.

**Conclusions:**

This is the first known study to investigate the associations between overall behavioral determinants and participant intentions to use a parenting chatbot in a low-income setting. This study identifies behavioral determinants of engagement for improved delivery of DHIs, considering the need to provide low-cost, scalable parenting support through digital platforms that engage parents, especially those in low-income contexts. Future research should explore methods to investigate mechanisms that regulate behavior to enhance the development of DHIs.

## Introduction

### Background

Research demonstrates the benefits of delivering public health interventions through digital platforms. In recent years, chatbots have been used to address structural barriers to accessing evidence-based interventions delivered through in-person programs [1,2], with increasing evidence suggesting their effectiveness in improving a range of health and behavioral outcomes, including mental well-being [3], supporting weight loss [4,5], and positive parenting practices [6]. While digital innovation offers a potentially cost-effective means to deliver public health programs at scale within low-income settings, user engagement rates are typically low [7]. Some of the identified critical barriers to participant engagement in digital interventions include the difficulty of use, lack of culturally adapted content (including human-like illustrations), low levels of digital literacy, and socioeconomic disadvantage—all of which can potentially exacerbate inequality when adapting digital alternatives from in-person programs [6,8,9].

Engagement in digital health interventions (DHIs) is theorized to be influenced by various factors or behavioral determinants. These include enjoyment, motivation, aesthetics and design, personal relevance, and ease of use [10,11]. We define “Behavioral Determinants of Engagement” as any factor characterized by attitudes, ideologies, and belief systems of the individual and the socioeconomic environment that influences the use of digital applications for behavior change. Even though behavioral determinants of health have been broadly studied and have been shown to influence behavior change [12,13], there is a gap in understanding the role of behavioral determinants on engagement in DHIs implemented in low- and middle-income country settings. The study of behavioral determinants in DHIs provides a broader approach to investigating the pathways leading to engagement by helping to identify individual and societal characteristics, such as socioeconomic status, sex, age, and geographic location, that may facilitate users’ uptake. Previous studies have evaluated user acceptance of DHIs by investigating participants’ (1) intention to use [14] and (2) engagement (ie, actual use) of the health technology [15]. Although actual engagement may suggest user acceptance of DHIs, understanding predictors of intention to use is an important predecessor of engagement in many behavior change theoretical models.

### Measuring the Behavioral Determinants of Engagement

We combined the following models to develop a Digital Health Engagement Model (DHEM) that uses a multilevel approach to study how behavioral determinants predict intention as well as moderate the relationship between intention to use and engagement with DHIs: the Theory of Planned Behavior, the Technology Acceptance Model, and the PEN-3 Model. Ajzen’s Theory of Planned Behavior [16] explains how personal beliefs, namely attitude (ie, positive or negative beliefs about potential outcomes of the desired behavior), subjective norms (ie, normative expectations of others related to enrolling in the desired behavior), and perceived control (ie, individual’s perceptions of the ease of difficulty in performing the desired behavior), predict and explain behavior of individuals. Davis’s Technology Acceptance Model [17] suggests that intention is determined by an individual attitude toward using a technology, which is mediated by perceived usefulness (ie, the achievement of job-related or personal goals due to the usage of the technology) and perceived ease of use (ie, how flexible, simple, and compatible the technology is) [18,19].

We drew from the relationships and expectations domain of the PEN-3 model to conceptually map predictors of intention retrieved from the Theory of Planned Behavior, the Technology Acceptance Model, and previous literature on digital health studies. We labeled those factors as behavioral determinants of engagement. Airhihenbuwa’s PEN-3 Model foregrounds the role of cultural factors in developing public health interventions and incorporates three domains: cultural empowerment, cultural identity, and relationships and expectations [20]. The domain of relationships and expectations includes the constructs of perceptions, nurturers, and enablers. In the context of DHIs, perceptions can be positive if they contribute to an individual’s active engagement with it (eg, users may perceive the DHI as a reliable source for finding health information) and negative if they negatively affect their active engagement with the DHI (eg, users may perceive that it is hard to manipulate the DHI) [18,19]. Similarly, positive enablers are the institutional resources encouraging the use of DHIs, such as clearly engaged marketing materials, broad network coverage, and external human support (eg, in-person or remote staff coaching for health technology use and troubleshooting support) [21-23]. On the other hand, negative enablers may include unsupportive structures for an individual’s access and use of DHI, such as poor network coverage and low access to cell phones and electricity. Nurturers are positive when an individual has family or peer support in the use of DHI or negative when unsupportive (eg, a user may be discouraged from using a parenting chatbot due to the negative opinions of their family members) [24].

### The DHEM

**Figure 1.**
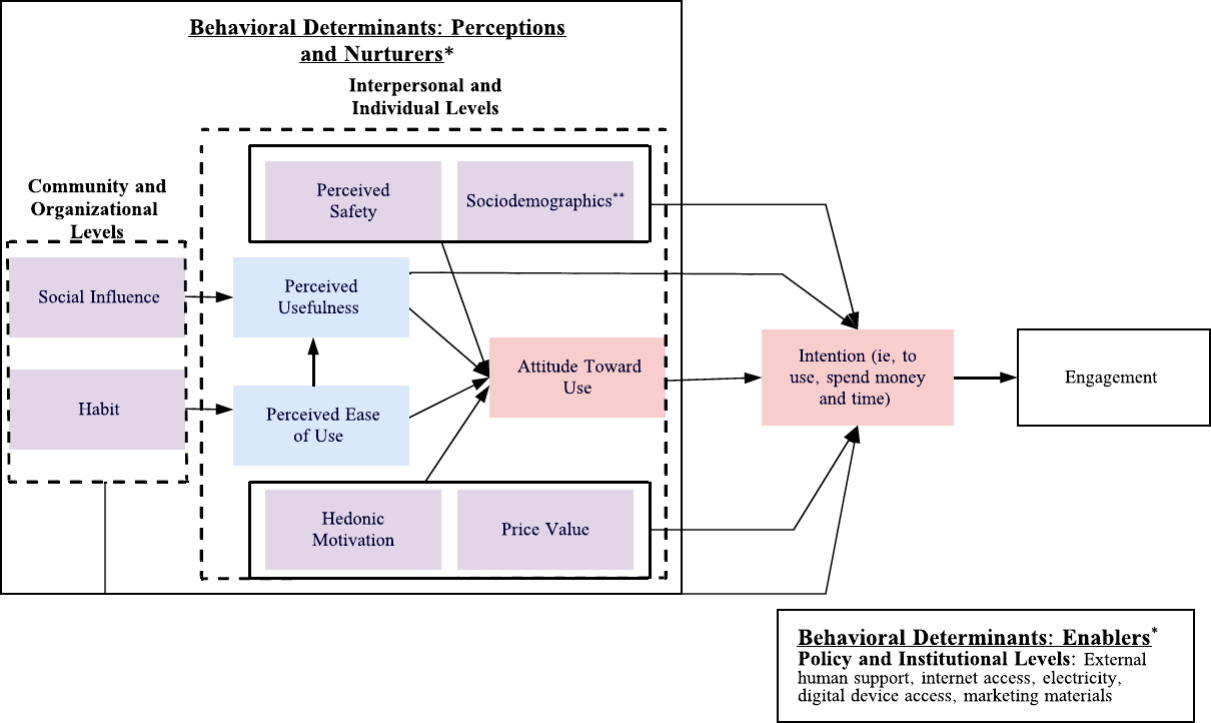
The Digital Health Engagement Model. The blue boxes indicate variables that belong solely to the Technology Acceptance Model, the pink boxes indicate variables that belong to the Technology Acceptance Model and Theory of Planned Behavior, and the purple boxes indicate the external variables. The “*” indicates variables mapped under the Perceptions, Nurturers, and Enablers constructs of the PEN-3 model, while “**” indicates sociodemographics such as age, gender, income, education level, and digital literacy.

The DHEM combines these theoretical frameworks by mapping behavioral determinants at three levels: (1) individual and interpersonal (eg, knowledge, attitudes, and beliefs affecting individual motivation toward the use of a DHI), (2) community and organizational (eg, beliefs and attitudes of close persons or groups toward the use of a DHI), and (3) policy and institutional levels (eg, resources within society and government promoting the use of DHI). In the DHEM, predictors of intention at the individual and interpersonal levels are perceived safety (ie, users’ belief that their personal information is safe when accessing health technology), perceived usefulness, perceived ease of use, hedonic motivation (ie, enjoyment of the health technology), price value (eg, cost of health technology or network connection), attitude, and sociodemographics (eg, age, gender, income, education level, digital literacy). Predictors at the community and organizational levels are social influence (ie, user-perceived support from peers and family to use a DHI) and habit (eg, frequency of using digital technologies for communication and access to public services). In contrast, predictors at the policy and institutional levels are external human support, internet access, electricity, and digital device access (Figure 1).

To guide the understanding of how the multidimensional aspects of intention may yield certain behavior outputs, the DHEM also incorporates a three-dimensional investigation of intention: (1) intention to use in the future, (2) intention to spend money (ie, pay to have access to DHI or network to use DHI), and (3) time to use in the future. The literature demonstrates that the intention–behavior relationship is dependent on time and contextual factors [25-27]. Therefore, in a low- and middle-income context where time and money constraints are often a reality, the three-dimensional study provides essential implications for advancing strategies for behavior change interventions, including those delivered digitally.

### South African Context

South Africa makes for an interesting case study for exploring user engagement, given the growing presence of DHIs across its evolving digital landscape. There is promising evidence on the feasibility of implementing chatbot-led DHIs in the country [28,29]. In 2022, an estimated 70% of South Africa’s population had internet access, and there were 162 mobile phone subscriptions per 100 people [30]. While chatbot-led public health interventions focused on health promotion and intimate partner violence prevention have been implemented in South Africa [28,29], evaluations of these interventions have documented no engagement rates. For instance, a South African chatbot-led study on intimate partner violence prevention reported no measure of user engagement [29].

Chatbot-led interventions, including those designed to promote positive parenting practices, have the potential to accelerate South Africa’s progress toward achieving the United Nations Sustainable Development Goals 4 and 16 for children (such as increasing access to education, safety, responsive caring, and violence against children prevention strategies) [31]. Even though in-person parenting programs previously implemented in South Africa have been shown to effectively target family factors (such as family relationship quality, abuse, and neglect) that influence adolescents’ health and well-being and reduce the risk for early pregnancy and HIV infection [32,33], they remain difficult to scale at a national level. While low-cost, chatbot-led interventions offer a promising way to scale parenting programs, challenges related to user engagement and the limited research explicitly focused on this issue make it difficult to provide parenting support among those who may not be able to enroll in person due to access barriers such as employment, transportation, and childcare costs.

### Study Objectives

The objectives of this study are as follows: first, to explore the factor structure for a locally adapted measurement of behavioral determinants; second, to examine how behavioral determinants of engagement are associated with the intention to use a chatbot delivered to parents and caregivers of adolescent girls as part of a hybrid-digital parenting intervention in South African low-income communities; and third, to investigate the moderating effect of individual-level sociodemographic and psychosocial characteristics on associations between determinants and intentions.

## Methods

### Study Design and Setting

This cross-sectional study was nested within a 2×2 clustered factorial trial (clusters were assigned to receive ParentText alone or combined with in-person sessions and a facilitated WhatsApp support group) that followed the Multiphase Optimization Strategy principles [34]. The trial aimed to optimize user engagement in the chatbot component of a hybrid digital parenting program developed by Parenting for Lifelong Health [34]. The program was implemented by mothers2mothers (m2m), a South African non-profit organization, as part of a large-scale HIV-prevention project “Children and Adolescents Are My Priority” (CHAMP) [34]. The CHAMP project was funded by the USAID (United States Agency for International Development) PEPFAR (US President's Emergency Plan for AIDS Relief) DREAMS (Determined, Resilient, Empowered, AIDS-free, Mentored, and Safe) Initiative, which focuses on strengthening family relationships and communications and providing biomedical prevention resources and HIV treatment to 28,500 adolescent girls, aged 10 and 19 years, and young women [35]. This study was delivered from August to December 2023 across municipalities in the Ehlanzeni and Nkangala Districts of Mpumalanga Province, South Africa. Mpumalanga is characterized by high literacy and high unemployment [36], and the areas in which the trial was implemented were mostly rural and peri-urban zones, with agriculture, fishing, and mining as the primary economic sectors [37].

### Eligibility Criteria

Caregivers recruited from the CHAMP community project were eligible if they (1) were aged 18 years or older and spoke English, isiZulu, or siSwati, (2) were caring for an adolescent girl aged between 10 and 17 years, (3) were already enrolled in CHAMP, (4) were living in the same household with the adolescent girl for a minimum of four nights over the previous 3 months, (5) had access to a mobile phone compatible with WhatsApp, (6) were willing to provide written informed consent to participate in the full study, and (7) were willing to enroll in the parenting chatbot and receive messages via WhatsApp. Caregivers with severe learning disabilities or mental health disorders were ineligible.

### Data Collection Procedures

Data collection was conducted by trained m2m facilitators who were also responsible for recruiting participants into the trial and delivering the intervention. Baseline data were collected from caregivers and adolescent girls at community settings. Facilitators led participants through the chatbot registration and first parenting module (ie, approximately 10 min of user interaction). After completion of onboarding activities, caregivers completed the behavioral determinants and intention to use the chatbot survey. Data from paper-based questionnaires were abstracted by trained data capturers using Open Data Kit for analysis.

### Digital Health Intervention

**Figure 2.**
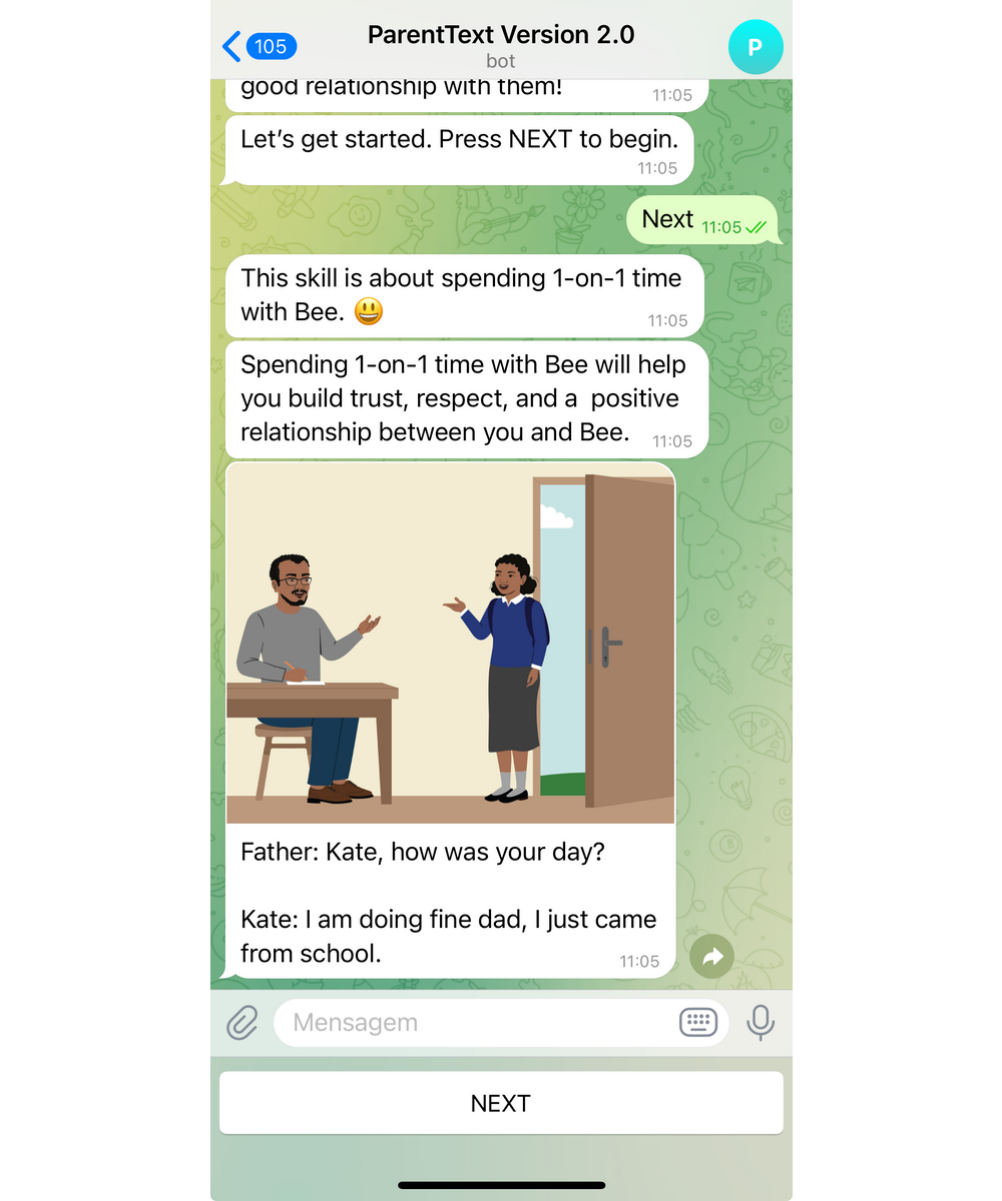
A display of a vignette in ParentText on a smartphone screen.

ParentText is a mobile rule-based chatbot adapted from the Parenting for Lifelong Health suite of programs to address barriers to access and scale of in-person programs [34]. It delivers personalized and gamified scheduled and on-demand messages through text, audio, and visual messages using UNICEF RapidPro, an open-source platform for building interactive messaging systems and delivered through social messaging apps such as WhatsApp (see Figure 2). While the overall content package includes messages based on development stages for caregivers of children aged 0‐23 months, 2‐9 years, and 10‐17 years, this study only used the content targeting caregivers of children aged 10-17 years. ParentText delivers content over nine positive parenting goals composed of 37 learning modules aiming to improve parent–adolescent communication (Figure 2), improve engagement in learning, improve adolescent mental health, and reduce risky behaviors by establishing a healthy routine with clear instructions and rules [34]. Modules are described in Table S1 in Multimedia Appendix 1.

Initial piloting of ParentText in South Africa found low participant engagement, with the reported proportion of participants accessing the content delivered across four topics ranging from 20% (20/96) to 28% (27/96) of the sample [38]. Based on results from the pilot evaluation, the chatbot was subsequently revised to enhance the user experience by incorporating personalization features and adding tools to improve usability and support ongoing engagement [39]. ParentText is also being tested in the preschool system in Malaysia and the social welfare system in Mexico [40]. Delivery in South Africa was intended to support positive parent–adolescent relationships and prevent HIV risk behaviors as a key component of the CHAMP project.

### Measures of Behavioral Determinants

Existing literature and the DHEM guided the instrument development used to measure behavioral determinants and intention to use. While previous studies have identified associations between behavioral determinants (ie, perceived usefulness, perceived ease of use, attitude, hedonic motivation, habit, price value, and social influence) with intention to use [7,41-43], we found no existing questionnaires that specifically assess the behavioral determinants of potential users in the context of a chatbot-led parenting intervention. For this reason, we developed a questionnaire to measure caregivers’ perceptions specific to the chatbot and their general usage frequency of WhatsApp, the platform on which ParentText was hosted. Given WhatsApp’s role as a communication tool, caregivers’ frequency of use can offer insights into its usability within their social networks (eg, family, neighbors, and friends).

The behavioral determinants and intention to use questionnaires consisted of 16 questions that were tested with the local research team for improved contextualization and translation. The outcome intention to use was assessed using two separate items that asked caregivers about their intention to use ParentText in the future and their intention to spend their mobile data to use ParentText. Both items were rated on a Likert scale ranging from 0 (strongly disagree) to 4 (strongly agree). Behavioral determinants were assessed using 14 items across seven domains derived from the Technology Acceptance Model and its extensions [17,19]. Perceived ease of use was measured using two items assessing caregivers’ perceived understanding and capacity to interact with the chatbot. Perceived usefulness was measured using seven items (eg, “I think that ParentText would help me learn how to manage my child’s behavior”). The remaining five domains, attitude toward use, hedonic motivation, price value, habit, and social influence, were each measured using a single item. All 16 items were rated on a Likert scale ranging from 0 (strongly disagree) to 4 (strongly agree). The items are described in Table S2 in Multimedia Appendix 2.

Covariates such as age, gender, and financial efficacy (ie, how confident caregivers are in their ability to manage their finances) were also assessed prior to administering the determinants and intention questionnaire. The choice of covariates was based on previous research reporting that age, gender, and financial status are associated with users’ intentions to use DHIs [44-46]. In all questions, participants were given the option to select the response, “I do not want to answer.” All measures (Table S2 in Multimedia Appendix 2) and ParentText content were translated from English into isiZulu and siSwati, the local home languages spoken by participants adopting back-translation procedures.

### Moderators

Moderators included age, absence of the father in the house, financial efficacy, anxiety, depression, and stress. Age was measured in years, and the absence of the father in the house was a binary variable (yes and no). Financial efficacy was measured on a scale ranging from “not at all true” (0) to “exactly true” (3). Stress was measured using the parental stressors subscale (6 items) of the Parental Stress Scale on a scale of 0 to 20 and anxiety and depression using the 4-item Patient Health Questionnaire on a scale ranging from 0 to 12 [47,48].

### Statistical Analyses

Data analysis was conducted using RStudio (R version 4.2.2) and Visual Studio Code (Python version 3.10.6) software. Missing data accounted for a maximum of 10% for predictor (n=91) and outcome (n=108) variables and was replaced using multiple imputation-chained equations following Graham and colleagues’ recommendations [49]. Imputation was conducted using fully conditional specifications, predictive mean matching for numeric data, and polytomous regression for multicategory data. Twenty interactions were used to refine imputations, with five imputations created accounting for clustered standard errors. Beesley’s stacked approach was conducted on the imputed datasets for weighing models and clustered standard errors to account for the between-cluster variance across the 32 clusters in the study [50]. Descriptive data was presented using frequencies and percentages for categorical variables and means and standard deviations for numeric variables.

To explore the factor structure of the behavior determinants measure, reliability analysis was performed on the extracted factors using Cronbach α and omega coefficients to assess the overall consistency, with *α*>.70 representing high reliability, .35>α<.70 representing medium reliability, and *α*<.35 representing low reliability [51]. A collinearity test was conducted using Pearson’s correlations and variance inflation factor (VIF), with VIF <1 representing no multicollinearity, 1>VIF <5 representing moderate collinearity, and VIF >5 representing high multicollinearity [52]. We also conducted principal component (PC) analysis to ensure that factors in the overall behavioral determinants composite construct contributed equally to it. PC analysis used individual scores of five factors (ie, attitude, hedonic motivation, habit, price value, and social influence) and the perceived usefulness (from 0 to 28) and the perceived ease of use (goes from 0 to 8) aggregate scores.

Next, to investigate how behavioral determinants and intentions to use are associated, ordered logistic regression models were applied to test for the predictive significance of behavioral determinants on both outcomes assessing caregiver intention to use ParentText. All four models (multivariable models with individual behavioral determinants and univariate models with the composite construct) included covariate adjustments for age, gender, and financial efficacy to account for confounding effects. Finally, to test the moderating effect of individual-level sociodemographic and psychosocial characteristics (ie, caregiver age, presence of the father in the house, financial efficacy, caregiver anxiety and depression, and caregiver stress), models with interactions between moderator and overall behavioral determinants construct were conducted. Results reports followed the Consolidated Standards of Reporting Trials of Electronic and Mobile Health Applications and Online Telehealth (CONSORT-EHEALTH) checklist [53].

### Ethical Considerations

Ethics approval for the study was granted by the University of Cape Town Center for Social Science Research Ethics Committee (reference: CSSR 2023/05), the University of Oxford Social Sciences and Humanities Interdivisional Research Ethics Committee (reference: R88177/RE001), and the Mpumalanga Department of Health and Department of Social Development (reference: R69569/RE003) [34]. Participants provided written informed consent prior to completing paper-based baseline surveys and were offered 50 ZAR internet bundles (approximately US $2.60) as an incentive to participate in the study [34]. Deidentified data sets were stored securely in a password-protected server at the University of Oxford and paper-based surveys stored by the m2m South Africa office in compliance with the South African Protection of Personal Information Act [34].

## Results

**Figure 3.**
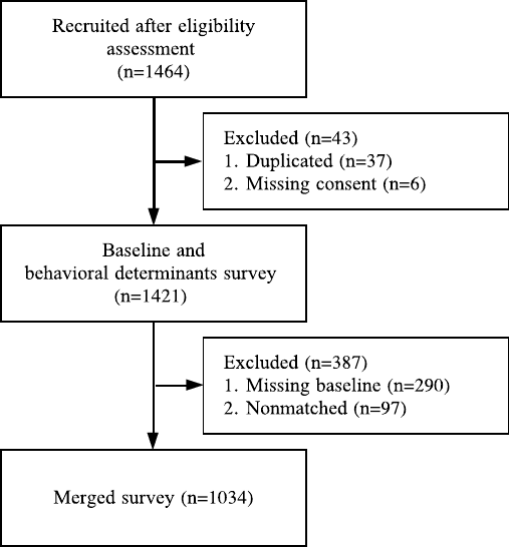
Study recruitment.

Out of the 1464 caregivers recruited for eligibility, 1034 were included in this study with matched baseline demographic and behavioral determinant surveys (Figure 3).

### Caregiver Baseline Characteristics

Participating caregivers were predominantly female (94%, 974/1034), and the average age was 38 years (SD 9). Almost two-thirds reported that they were in a relationship (64%, 665/1034). Reported paternal absence rates were 51% (537/1034), which were slightly below the national South African average (58%) [47]. Most caregivers were the biological parent (81%, 847/1034), and 10% (125/1034) reported that the adolescent girl in their care was a paternal orphan (Table 1).

**Table 1.** Baseline characteristics of participants (N=1034).

Characteristic	Value
Caregiver age, mean (SD)	38 (9)
Place of residence, n (%)	
Ehlanzeni	716 (69)
Nkangala	318 (30)
Caregiver gender, n (%)	
Female	974 (94)
Caregiver status, n (%)	
Biological parent	847 (81)
Relationship status, n (%)	
Partnered^a^	665 (64)
Financial efficacy, mean (SD)^b^	1.64/3 (1.09)
Absence of father in the house, n (%)^c^	537 (51)
Caregiver disability, n (%)	
Yes^d^	76 (7)
Child age, mean (SD)	12 (2.00)
Child gender, n (%)	
Female	1,034 (100.0)
Adolescent school enrollment (ie, school year), n (%)	
Grade 4‐9	867 (83)
Grade 10‐12	167 (16)
Caregiver anxiety and depression, mean (SD)	3.39/12 (3.21)
Caregiver stress, mean (SD)	8.60/20 (4.21)

a Partnered refers to married, civil, or noncivil partnership status.

b Higher values imply greater capacity to manage finances.

c Families where the father passed away or lives elsewhere.

d Includes caregiver-reported cognitive, learning, auditory, visual, physical (any motor limitations and difficulties in performing self-care activities such as getting dressed), and functional disabilities (any pre-existing non-communicable health conditions such as diabetes, cardiovascular disease, and cancer).

### Descriptives of Behavioral Determinants and Intention

Caregivers reported a mean score of 2.99 (SD 0.55) out of 4 on their perceived usefulness of ParentText, a mean of 3.02 (SD 0.67) on their enjoyment, and a mean of 2.86 (SD 0.77) on perceived ease to interact with the chatbot. They also reported a mean of 2.91 (SD 0.70) on their perceptions that their peers and family would support them using ParentText and a mean of 2.81 (SD 0.78) on how worthwhile they believed it was to spend mobile data on the chatbot. The mean attitude of caregivers toward DHIs such as ParentText was 3.05 (SD 0.68) and WhatsApp usage habits was 2.76 (SD 0.85). Respondents also reported a mean of 2.85 (SD 0.79) and 2.90 (SD 0.72) regarding their intention to use their mobile data and to continue using ParentText in the future, respectively.

### Psychometric Properties of Behavioral Determinants Measure

#### Principal Component Analysis

Results indicate that all seven items loaded relatively equally on the first PC, which accounted for 52% of the total variance of the overall behavioral determinant construct (52% PC1, 13% PC2, 9% PC3, 7% PC4, 6% PC5, 5% PC6, and 4% PC7). Using the scores from PC1, a summary composite score across all four subscales ranging from 0.67 to 18.64 was generated and used in the regression models (Table S3 in Multimedia Appendix 3).

#### Reliability Tests

The overall reliability of the instrument exceeded the acceptable threshold (*α*=.90; 95% CI 0.89‐0.90; *Ω*=0.91). Reliability results for perceived usefulness, as measured by the *α* and omega coefficients, were also above 0.70 and thus considered high (Table S4 in Multimedia Appendix 3).

### Association Between Behavioral Determinants and Intention to Use

Higher levels of overall behavioral determinants were positively associated with higher caregiver intention to use ParentText (intention to use in the future: odds ratio [OR] 1.85, 95% CI 1.81‐1.90 and intention to use mobile data: OR 1.76, 95% CI 1.72‐1.81; Tables2  3). Individual behavioral determinant measures (ie, perceived usefulness and ease of use, attitude, hedonic motivation, habit) also emerged as positive predictors of both intention outcomes (Tables2  3). Price value and perceived ease of use were not significantly associated with intention to use, while social influence was not significantly associated with intention to spend mobile data. The results of goodness-of-fit (Table S6 in Multimedia Appendix 3) indicate pseudo-*R*^2^ values ranging from 17% to 37% in the overall behavioral determinants model and from 16% to 33% in the models with individual behavioral determinants, suggesting that the respective changes in the caregiver’s intentions to use ParentText are well explained by all predictors. VIF indicated collinearity ranging from 1.02 to 2.95 (Table S5 in Multimedia Appendix 3). Tables S7 and S8 in Multimedia Appendix 3 report the results of multivariable logistic models conducted on unimputed data for sensitivity analysis.

**Table 2.** Multivariable logistic regression results testing associations between behavioral determinants and intention to use ParentText in the future^a^.

Independent variable	OR^b^	95% CI	*P* value
Overall behavioral determinants	1.85 (0.01)	1.81‐1.90	<.001
Perceived usefulness	1.10 (0.00)	1.08‐1.12	<.001
Perceived ease of use	1.00 (0.02)	0.96‐1.04	.99
Attitude	1.67 (0.03)	1.60‐1.75	<.001
Hedonic motivation	1.77 (0.05)	1.66‐1.87	<.001
Habit	1.25 (0.03)	1.18‐1.31	<.001
Price value	1.02 (0.03)	0.97‐1.08	.34
Social influence	2.08 (0.12)	1.85‐2.34	<.001

a Note: All models included covariate adjustments for age, gender, and financial efficacy. Clustered standard errors in parentheses.

b Odds ratio.

**Table 3.** Multivariable logistic regression results testing associations between behavioral determinants and intention to spend mobile data to use ParentText in the future^a^.

Independent variable	OR^b^	95% CI	*P* value
Overall behavioral determinants	1.76 (0.01)	1.72‐1.81	<.001
Perceived usefulness	1.07 (0.00)	1.05‐1.08	<.001
Perceived ease of use	1.05 (0.02)	1.01‐1.09	<.001
Attitude	2.14 (0.06)	2.01‐2.27	<.001
Hedonic motivation	1.26 (0.03)	1.19‐1.33	<.001
Habit	1.17 (0.05)	1.08‐1.28	<.001
Price value	2.15 (0.10)	1.97‐2.36	<.001
Social influence	1.05 (0.03)	0.99‐1.11	.05

a Note: All models included covariate adjustments for age, gender, and financial efficacy. Clustered standard errors in parentheses.

b Odds ratio.

### Moderator Analyses

Moderator analysis suggested more relevance of behavioral determinants in predicting caregivers’ intention to use in families where fathers were absent (OR 2.03, 95% CI 1.79‐2.41) than those where fathers were present. A greater relevance of behavioral determinants in predicting caregivers’ intention to use was observed among caregivers experiencing higher levels of stress compared to those with lower stress levels (OR 1.09, 95% CI 1.05‐1.14). Behavioral determinants were also more relevant in predicting intentions to spend mobile data among older caregivers in comparison to younger caregivers (OR 1.04, 95% CI 1.03‐1.05), financially stressed families in comparison to those more financially stable (OR 0.38, 95% CI 0.30‐0.48), and in families where fathers were absent in comparison to those where fathers were present (OR 2.34, 95% CI 2.01‐2.72). No moderating effects were observed on baseline levels of caregiver anxiety and depression (Table 4).

**Table 4.** Results of the interaction effect of sociodemographic and psychosocial characteristics on the associations between behavioral determinants and intention to use^a^.

Moderator	Outcome	^b^OR	95% CI	*P* value
Age	Intention to use in future	1.00 (0.00)	0.99‐1.00	.82
	Intention to spend mobile data	1.04 (0.00)	1.03‐1.05	<.001
Absence of father in the house^c^	Intention to use in future	2.03 (0.07)	1.79‐2.41	<.001
	Intention to spend mobile data	2.34 (0.10)	2.01‐2.72	<.001
Financial efficacy	Intention to use in future	0.90 (0.12)	0.71‐1.15	.43
	Intention to spend mobile data	0.38 (0.12)	0.30‐0.48	<.001
Anxiety and depression	Intention to use in future	1.02 (0.04)	0.94‐1.10	.59
	Intention to spend mobile data	0.96 (0.03)	0.89‐1.02	.25
Stress	Intention to use in future	1.09 (0.02)	1.05‐1.14	<.001
	Intention to spend mobile data	0.91 (0.03)	0.86‐0.97	.007

a Note: Clustered standard errors in parentheses. Financial Efficacy range 0‐3. Anxiety and depression range 0‐12. Stress range 0‐20.

b Odds ratio.

c Reference category: Present father.

## Discussion

### Evidence From the Theoretical Framework

Our findings highlight the positive associations between overall behavioral determinants and caregivers’ intention to use the parenting chatbot, underscoring the strength of the composite construct in effectively capturing the seven underlying domains. For each one-unit increase in the overall behavioral determinants score, the odds of caregivers intending to spend mobile data on ParentText were 76% higher and the odds of intending to use ParentText in the future were 85% higher, compared to caregivers with lower scores. Similarly, individual behavioral determinant domains were also associated with intention, offering more nuanced insights into the context in which the chatbot was implemented.

**Figure 4.**
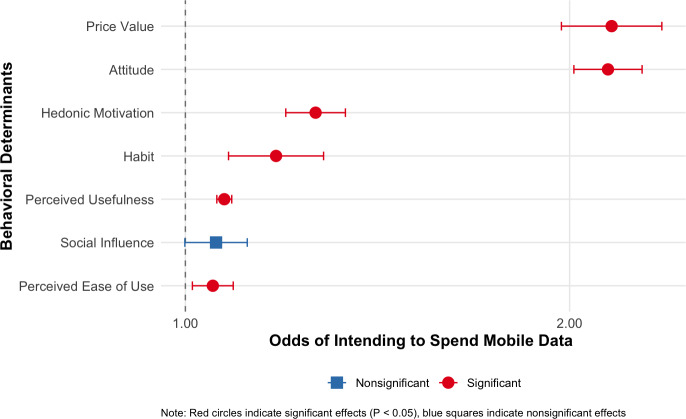
Forest plot of logistic regression coefficients testing associations between behavioral determinants and intention to spend mobile data to use ParentText in the future.

Results demonstrated that perceived usefulness, attitude, and hedonic motivation were positively associated with intention to use, aligning with theory and with findings from previous research that reported such factors as positive predictors of intention to use [54,55]. Findings indicating positive associations between price value (ie, the perception that the chatbot was worth spending mobile data on) and intention to spend mobile data (Figure 4) warrant further discussion. Participants’ prior knowledge that they would receive internet bundles to use ParentText may have influenced these observed positive associations. In low-income contexts such as rural and peri-urban South Africa, perceiving the value of ParentText and having a positive attitude toward DHIs may have influenced the intention of caregivers to use their data to interact with the chatbot instead of spending it on something else. There is ambiguity on the associations of financial cost to accessing a DHI with participants' intention to use, as some studies report negative [44,56], while others report no associations [57]. Our findings suggest that access to the internet, which, in the context of our study, has financial implications, may increase the acceptance of DHIs within low-income households.

**Figure 5.**
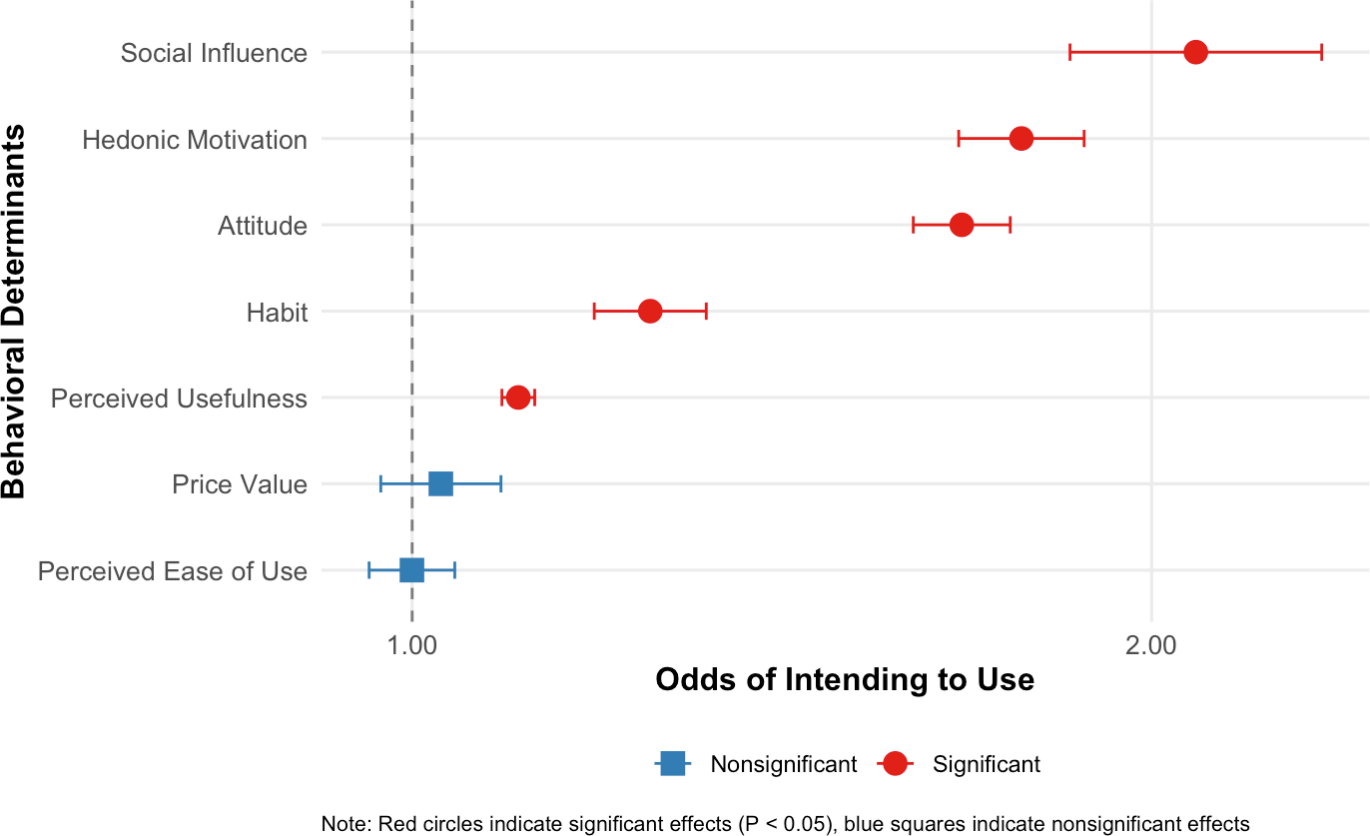
Forest plot of logistic regression coefficients testing associations between behavioral determinants and intention to use ParentText in the future.

Social influence, found in previous research to be associated with intention to use a DHI [57,58], was also positively associated with caregivers’ intention to use ParentText (Figure 5), as measured by agreement with the statement, “People who are important to me think that digital tools such as ParentText could support me as a parent.” In this study, participants responded to the questionnaire while in a group setting during onboarding, during which they may have been aware of other caregivers’ intentions toward using ParentText. Those factors may have positively influenced caregivers’ impressions that their family and peers would support their usage of ParentText. In addition, results indicating positive associations between WhatsApp usage habits and intentions to use ParentText suggest the potential efficacy of adopting the most used and familiar communication platforms among potential DHI users to increase user acceptance.

### Implication of Results in the South African Context

Our findings suggest that caregivers in South Africa who were introduced to the ParentText chatbot had positive intentions to continue to use it. Overall behavioral determinants became more relevant in predicting caregivers’ intention to spend mobile data among families with older caregivers, families where the father was absent, and families that were less financially stable. These findings suggest that families with older caregivers, without fathers, or with greater levels of financial stress may require more structural support to engage in DHIs than those with younger caregivers, where fathers are present, and that are more financially stable. Therefore, there is great value in engaging fathers when delivering parenting chatbots, as previous research has reported their important role in families’ health and well-being [59].

### Study Strengths and Limitations

This study contributes to advancing the cycle of better development and implementation of DHIs by introducing a framework and a composite construct that explains the holistic role of behavioral determinants of engagement. In contexts where collecting data on all individual behavioral determinants is not feasible, the composite construct provides a practical alternative, allowing for the use of fewer items while still effectively capturing the multidimensional factors that influence users’ intentions to engage with DHIs. Even though we could only investigate individual- and interpersonal-level factors and caregivers’ personal impressions of community-level factors, their integration in the development and implementation stages can support good practices and help maximize DHI uptake.

Several limitations should be considered when interpreting our findings. First, selection bias may have influenced the results, as participants were caregivers already enrolled in the CHAMP program and owned WhatsApp-enabled phones, potentially representing a tech-savvy and intervention-positive subset of the population. Second, intention scores might have been skewed, as participants may have reported more positive intentions due to the provision of internet bundles, thereby confounding our results. Third, the cross-sectional design limited our ability to examine causal relationships between behavioral determinants and intention to use. Fourth, the behavioral determinants measurement did not capture policy-level enablers (eg, internet access, electricity), nor did we measure the participant’s intention to spend time on the chatbot. Lastly, while we conducted face validity and tested the reliability of the behavioral determinant measures, we were unable to assess other types of validity due to the limited capacity of our research team.

### Future Directions

Future research could employ a longitudinal or experimental design to investigate the relationships between behavioral determinants and intentions. It should also investigate how behavioral determinants and intentions are associated with engagement because a broader understanding of this pathway greatly supports efforts to improve the deployment of DHIs. Studies should also assess the construct, criterion, and content validity of the behavioral determinants and intention measurements and investigate the factors at the policy and institutional levels. Lastly, it is also essential to further investigate factors that are associated with positive intentions toward DHIs among those facing stress.

### Conclusions

To the best of our knowledge, this is the first study to investigate how behavioral determinants of engagement are associated with the intention to use a parenting chatbot delivered in a low- and middle-income context. In conclusion, we recommend that developers and implementation scientists emphasize the value and quality of the DHIs while targeting aspects that enhance receptivity to social influence. Efforts to minimize network connection costs could also be provided through internet hotspots. The perception of affordability can trigger the sense that an individual is getting a good deal, which consequently leads to positive intentions. Similarly, implementation strategies that incorporate collaboration with community partners can foster positive normative influences toward DHIs, potentially leading to more favorable intentions toward use. The adoption of the most used communication platforms can also be prioritized to support positive intentions among potential users of chatbot-led interventions. While these early-stage findings suggest that chatbots may be effective platforms for overcoming barriers to accessing in-person programs in real-world settings, the generalizability of these results to other contexts remains uncertain.

## Supplementary material

10.2196/76992 Multimedia Appendix 1 Intervention modules: ParentText content.

10.2196/76992 Multimedia Appendix 2 Items measuring behavioral determinants and intention to use.

10.2196/76992 Multimedia Appendix 3 Supplementary analyses.

## Abbreviations

DHIs: digital health interventions
DHEM: Digital Health Engagement Model
CHAMP: Children and Adolescents Are My Priority
m2m: mothers2mothers
CONSORT-EHEALTH: Consolidated Standards of Reporting Trials of Electronic and Mobile Health Applications and Online Telehealth
VIF: variance inflation factor
