# Supplementary material for: Exploring the Association Between Behavioral Determinants and Intention to Use a Chatbot-Led Parenting Intervention by Caregivers of Adolescent Girls in South Africa: Cross-Sectional Study
Source: JMIR Pediatr Parent. 2025 Sep 22;8:e76992. doi: 10.2196/76992 (PMC12453451; doi:10.2196/76992)
Supplement: Multimedia Appendix 1 [file pediatrics-v8-e76992-s001.docx]

# Multimedia Appendix

Table S1. Intervention Modules: ParentText Content

| **Goal** | **Number of Modules** | **Content Overview** |
| --- | --- | --- |
| Improve My Relationship with my Teen | 3 | Techniques to spend one-on-one time with adolescents, praise and talk about feelings. |
| Understand Teen Development | 3 | Key knowledge to understand adolescent mental, social and physical changes. |
| Support My Teens Education | 5 | Techniques to create a fun and positive environment to support the academic learning process of adolescents. |
| Create Structure for my Teen | 4 | Techniques to establish a healthy routine for adolescents, give clear instructions and establish rules around the house and other academic and non-academic activities. |
| Manage my Teen’s Behavior | 4 | Skills to manage adolescents’ misbehavior. |
| Care for my Teen’s Wellbeing | 5 | Techniques to identify and manage stress signs and skills for supporting adolescents. |
| Keep my Teen Safe | 4 | Skills and strategies to keep adolescents safe in the community and relationships. |
| Improve my Family’s Finances | 4 | Skills to improve household budgeting through lessons related to savings and expenses. |
| Have a Healthy Relationship with my Partner | 5 | Techniques for solving conflicts and sharing responsibilities in marriage, civil or non-civil partnership. |
