# Supplementary material for: Exploring the Association Between Behavioral Determinants and Intention to Use a Chatbot-Led Parenting Intervention by Caregivers of Adolescent Girls in South Africa: Cross-Sectional Study
Source: JMIR Pediatr Parent. 2025 Sep 22;8:e76992. doi: 10.2196/76992 (PMC12453451; doi:10.2196/76992)
Supplement: Multimedia Appendix 2 [file pediatrics-v8-e76992-s002.docx]

Multimedia Appendix

Table S2. Items measuring behavioral determinants and intention to use

| **Outcomes** | **Survey Questions** |
| --- | --- |
| **Intention to use** | - I intend to use ParentText in the future. - Ngihlose ukusebenzisa i-ParentText esikhathini esizayo - Ngihlose kuphindze ngimsebentise ParentText   nasesikhatsini lesitako |
|  | - I plan to continue to use my mobile data to interact with ParentText. - Ngihlele ukuqhubeka nokusebenzisa idatha yeselula yami ngihlanganyele neParentText nsuku zonke - Ngihlela kuchubeka ngisebentise imali yelucingo lwami kusebentisa ParentText onkhe emalanga |
| **Behavioral Determinants of Engagement** | |
| Perceived ease of use | - I understand how to use ParentText. - Ngiyayiqonda indlela yokusebenzisa i-ParentText - Ngiyakucondza kumsebentisa ParentText |
|  | - I find ParentText easy to use. - Kulula ukuyisebenzisa i-ParentText - Ngikukhandze kulula kumusebentisa ParentText |
| Perceived usefulness | - I think that ParentText would help me learn how to manage my child’s behavior. - Ngicabanga ukuthi i-ParentText ingangisiza ngifunde ukuphatha ukuziphatha kwengane yami kangcono - Ngicabanga kutsi ParentText utangisita kungifundzisa kutsi ngibhekane nekutiphatsa kwemntfwana wami kancono |
|  | - I think that ParentText will help me support my child’s learning and development. - Ngicabanga ukuthi i-ParentText izongisiza ngisekele ukufunda nokuthuthuka kwengane yami - Ngicabanga kutsi ParentText utangisita kusekela umntfwanami kufundza nekukhulu |
|  | - I think that ParentText will help me build a more positive relationship with my child. - Ngicabanga ukuthi i-ParentText izongisiza ngakhe ubudlelwano obuhle nengane yami - Ngicabanga kutsi ParentText utangisita kuba nebudlelwane lobuhle nemntfwana wami |
|  | - I think that ParentText will help me reduce stress. - Ngicabanga ukuthi i-ParentText izongisiza ngehlise ingcindezi - Ngicabanga kutsi ParentText utangisita kwehlisa lizinga lekukhatsateka |
|  | - I think that ParentText will help me improve relations between me and my partner. - Ngicabanga ukuthi i-ParentText izongisiza ngithuthukise ubudlelwano phakathi kwami nomlingani wami - Ngicabanga kutsi ParentText utangisita ngitfutfukise budlelwane bami nalengitsandzana naye |
|  | - I think that ParentText will help me learn how to keep my child healthy and safe from dangerous situations in the community (ie, spend night away from home, use drugs, drink up with friends). - Ngicabanga ukuthi i-ParentText izongisiza ingifundise ukuthi ingane yami ngingayigcina kanjani iphilile ezimweni eziyingozi emphakathini (okungukuthi, ukuchitha ubusuku ungekho ekhaya, ukusebenzisa izidakamizwa, ukuphuza nabangane - Ngicabanga kutsi ParentText utangisita kungifundzisa kutsi ngibe nemntfwana lophilile futsi aphephile etimeni letinebungoti letivamise kwenteka emmangweni (lekufaka ekhatsi kungalali ekhaya,kusebentisa tidzaka miva nekunatsa tjwala nebangani) |
|  | - I think that ParentText will help me learn how to manage my finances better. - Ngicabanga ukuthi i-ParentText izongisiza ukuthi ngifunde ukuphatha imali yami kangcono - Ngiyacabanga kutsi ParentText utangisita ngikwati kuphatsa timali tami kancono |
| Attitude toward use | - Receiving daily messages from a chatbot like ParentText to support me as a parent is a good idea. - Ukuthola imilayezo yansuku zonke evela ku-chatbot efana ne-ParentText ukuze ngisekele njengomzali kuwumbono omuhle - Kutfola imilayeto ku chatbox malanga onkhe lefana neyaka ParentText kuyangesekela njengemtali futsi kungumcondvo lomuhle |
| Hedonic motivation | - I think that using ParentText is fun and enjoyable. - Ngicabanga ukuthi ukusebenzisa i-ParentText kumnandi futhi kuyajabulisa - Ngicabanga kutsi kusebentisa ParentText kulula futsi ngiyakutfokotela |
| Price value | - I think that ParentText will be worth using my data load for. - Ngicabanga ukuthi i-ParentText ikufanele ukuthi ngisebenzise amadatha wami kuyo - Ngicabanga kutsi ngitakujabulela kusebentisa ParentText ngemali leselucingweni lwami. Noma ngingeke |
| Habit | - I use WhatsApp a lot every day. - Ngisebenzisa i-WhatsApp kakhulu nsuku zonke - Ngiyamsebentisa WhatsApp kakhulu onkhe emalanga |
| Social influence | - People who are important to me think that digital tools such as ParentText could support me as a parent. - Abantu ababalulekile kimina bacabanga ukuthi amathuluzi edijithali afana ne-ParentText angangisekela njengomzali - Bantfu labamcoka kimi bacabanga kutsi kutfola lwati etincwadzini temoya letifaka ekhatsi ParentText kungangesekela njengemtali |
| **Demographics** | |
|  | Age, gender and financial efficacy |

Note: Questions are presented in English, isiZulu and siSwati
