# Supplementary material for: Exploring the Association Between Behavioral Determinants and Intention to Use a Chatbot-Led Parenting Intervention by Caregivers of Adolescent Girls in South Africa: Cross-Sectional Study
Source: JMIR Pediatr Parent. 2025 Sep 22;8:e76992. doi: 10.2196/76992 (PMC12453451; doi:10.2196/76992)
Supplement: Multimedia Appendix 3 [file pediatrics-v8-e76992-s003.docx]

## Multimedia Appendices

Table S3. Principal component scores

|  | PC1 | PC2 | PC3 | PC4 | PC5 | PC6 | PC7 |
| --- | --- | --- | --- | --- | --- | --- | --- |
| Perceived usefulness | 0.41 | -0.31 | 0.30 | -0.04 | 0.15 | 0.22 | -0.75 |
| Perceived ease of use | 0.37 | 0.14 | 0.75 | 0.18 | 0.16 | -0.15 | 0.42 |
| Attitude | 0.36 | 0.48 | -0.20 | -0.42 | 0.15 | 0.36 | 0.49 |
| Hedonic motivation | 0.39 | -0.29 | -0.10 | 0.07 | -0.70 | -0.48 | 0.01 |
| Habit | 0.36 | 0.51 | -0.13 | 0.21 | -0.38 | 0.62 | -0.01 |
| Price value | 0.33 | 0.53 | -0.14 | -0.64 | 0.14 | -0.35 | -0.12 |
| Social influence | 0.38 | 0.01 | -0.48 | 0.55 | 0.50 | -0.22 | 0.01 |

Table S4. Summary of behavioral determinants’ psychometric properties

| **Outcome** | **Number of items** | **M (SD)** | **Skew** | **Alpha coefficient** | **Omega coefficient** | **Spearman's**  **correlation** |
| --- | --- | --- | --- | --- | --- | --- |
| Overall reliability of behavioral determinants | 14 | 13.73 / 56 (2.40) | -1.33 | 0.90 (95% CI: 0.89-0.90) | 0.91 |  |
| Perceived usefulness (PU) | 7 | 15.00 / 28 (2.89) | -1.46 | 0.88 (95% CI: 0.87-0.89) | 0.93 |  |
| Perceived ease of use (PE) | 2 | 5.73 / 8 (1.55) | -1.55 | 0.84 (95% CI: 0.82-0.86) |  | 0.65 |
| Parental Stress Scale (PSS) | 6 | 8.60 / 20 (4.21) | -0.16 | 0.83 (95% CI: 0.81-0.84) | 0.88 |  |
| Patient Health Questionnaire (PHQ) | 4 | 3.39 /  12  (3.21) | 0.67 | 0.82 (95% CI: 0.80-0.84) | 0.84 |  |

Table S5. Summary of variance inflation factor test

|  | **INTENTION TO USE** | **INTENTION TO SPEND MOBILE DATA** |
| --- | --- | --- |
| **Variables** | **Variance Inflation Factor** | |
| Perceived Usefulness | 2.90 | 2.92 |
| Perceived Ease of Use | 2.95 | 2.93 |
| Attitude | 1.51 | 1.48 |
| Hedonic motivation | 1.49 | 1.57 |
| Habit | 1.73 | 1.67 |
| Price value | 1.57 | 1.49 |
| Social influence | 1.46 | 1.53 |

Table S6. Model summary for goodness of fit test for imputed data

|  | intention to use | | | intention to spend mOBILE DATA | | | | |  |
| --- | --- | --- | --- | --- | --- | --- | --- | --- | --- |
| INDEPENDENT  VARIABLE | Cox & Snell R² | Nagelkerke R² | McFadden R² | Cox & Snell R² | | Nagelkerke R² | | McFadden R² | |
| Overall behavioral determinants | 0.31 | 0.37 | 0.21 | 0.30 | 0.35 | | 0.17 | |  |
| Multivariable models | 0.27 | 0.33 | 0.18 | 0.29 | 0.33 | | 0.16 | |  |

Table S7. Multivariable logistic regression testing associations between behavioral determinants and intention to use for unimputed data

| INDEPENDENT  VARIABLE | ^a^ OR | ^b^ 95% CI | p-VALUE | ^a^ OR | ^b^ 95% CI | p-VALUE |
| --- | --- | --- | --- | --- | --- | --- |
|  | Intention to use | | | Intention to spend money on mobile data | | |
| Overall behavioral determinants | 1.93 (0.07) | 1.77 – 2.11 | <.001 | 1.91 (0.08) | 1.75 – 2.08 | <.001 |
| Perceived usefulness | 1.48 (0.05) | 1.32 – 1.67 | <.001 | 1.36 (0.06) | 1.20 – 1.53 | <.001 |
| Perceived ease of use | 1.15 (0.07) | 0.99 – 1.33 | .05 | 1.36 (0.17) | 1.14 – 1.61 | <.001 |
| Attitude | 0.89 (0.22) | 0.57 – 1.38 | .60 | 1.56 (0.25) | 1.09 – 2.24 | .01 |
| Hedonic motivation | 1.22 (0.21) | 0.80 – 1.85 | .33 | 0.77 (0.26) | 0.53 – 1.13 | .18 |
| Habit | 1.17 (0.16) | 0.84 – 1.63 | .32 | 1.07 (0.20) | 0.80 – 1.43 | .62 |
|  |  |  |  |  |  |  |
| Price value | 0.94 (0.16) | 0.68 – 1.31 | .75 | 2.10 (0.29) | 1.14 – 3.07 | <.001 |
| Social influence | 1.87 (0.19) | 1.27 – 2.74 | .001 | 0.87 (0.28) | 0.63 – 1.20 | .41 |

Table S8. Model summary for goodness of fit test for unimputed models

|  | intention to use | | | intention to spend mOBILE DATA | | | | |  |
| --- | --- | --- | --- | --- | --- | --- | --- | --- | --- |
| INDEPENDENT  VARIABLE | Cox & Snell R² | Nagelkerke R² | McFadden R² | Cox & Snell R² | | Nagelkerke R² | | McFadden R² | |
| Overall behavioral determinants | 0.36 | 0.43 | 0.25 | 0.37 | 0.42 | | 0.22 | |  |
| Multivariable models | 0.36 | 0.43 | 0.23 | 0.38 | 0.44 | | 0.23 | |  |
